# Scrotal Abscess Revealing Epididymal Tuberculosis: A Case Report and Literature Review

**DOI:** 10.7759/cureus.110641

**Published:** 2026-06-11

**Authors:** Aziz Lamghari, Omar Jendouzi, Soufiane Ait Essi, Hajar El Agouri

**Affiliations:** 1 Urology, Military Hospital Oued Eddahab, Agadir, Agadir, MAR; 2 Medicine and Pharmacy, Ibn Zohr University, Agadir, MAR; 3 Pathology, Military Hospital Oued Eddahab, Agadir, Agadir, MAR

**Keywords:** atypical epididymitis, complicated epididymitis, epididymal tuberculosis, genitourinary tuberculosis, scrotal abscess

## Abstract

Genitourinary tuberculosis is one of the most frequent forms of extrapulmonary tuberculosis and represents a significant health concern in endemic regions. Epididymal involvement is the most common manifestation of male genital tuberculosis. However, presentation as a scrotal abscess is uncommon and may lead to diagnostic confusion with other infectious conditions. We report the case of a 36-year-old man with no significant past medical or surgical history who presented with a two‑month history of progressive left scrotal pain associated with fever. The patient initially underwent drainage of a presumed scrotal abscess followed by conventional antibiotic therapy without clinical improvement. A second surgical exploration with debridement was required. Microbiological examination of pus revealed acid‑fast bacilli, while culture and polymerase chain reaction confirmed *Mycobacterium tuberculosis*. Histopathological examination of the excision specimen showed granulomatous inflammation with caseous necrosis, consistent with tuberculous infection. Epididymal tuberculosis should be considered in patients presenting with chronic scrotal abscess, particularly in tuberculosis‑endemic regions. Early microbiological investigation and appropriate anti‑tuberculous therapy are essential to avoid delayed diagnosis and complications.

## Introduction

Tuberculosis remains a major global health problem despite advances in diagnostic and therapeutic strategies. The World Health Organization (WHO) continues to report a high global burden, with millions of people developing tuberculosis each year and a substantial impact in low- and middle-income countries [1]. Although pulmonary disease is the most common presentation, extrapulmonary tuberculosis accounts for a meaningful proportion of cases and may involve almost any organ system [1].

Genitourinary tuberculosis (GUTB) is among the most frequent forms of extrapulmonary tuberculosis and is often under-recognized because symptoms may be absent or nonspecific [2-3]. Infection is usually secondary to hematogenous dissemination from a primary focus (often latent), and within the male genital tract, epididymal involvement is commonly reported [2-4].

Clinically, epididymal tuberculosis may present with chronic scrotal pain, scrotal swelling, or epididymal induration and may be mistaken for epididymo-orchitis or even a testicular tumor, leading to delayed diagnosis and inappropriate antibiotic treatment [2-4]. Less commonly, a scrotal or epididymal abscess may be the presenting symptom, further increasing diagnostic uncertainty and the likelihood of repeated surgical interventions before the underlying etiology is identified [5]. This rare presentation underscores the diagnostic challenge of epididymal tuberculosis and highlights the importance of considering it in cases of atypical or non-resolving scrotal infections. Early suspicion and prompt mycobacterial testing are essential to avoid diagnostic delay and improve patient outcomes.

We report a rare case of epididymal tuberculosis revealed by a scrotal abscess in a 36-year-old man managed at the Military Hospital Oued Eddahab in Agadir, Morocco. Morocco remains a tuberculosis-endemic country, where extrapulmonary forms continue to be encountered in routine clinical practice.

## Case presentation

**Figure 1:**
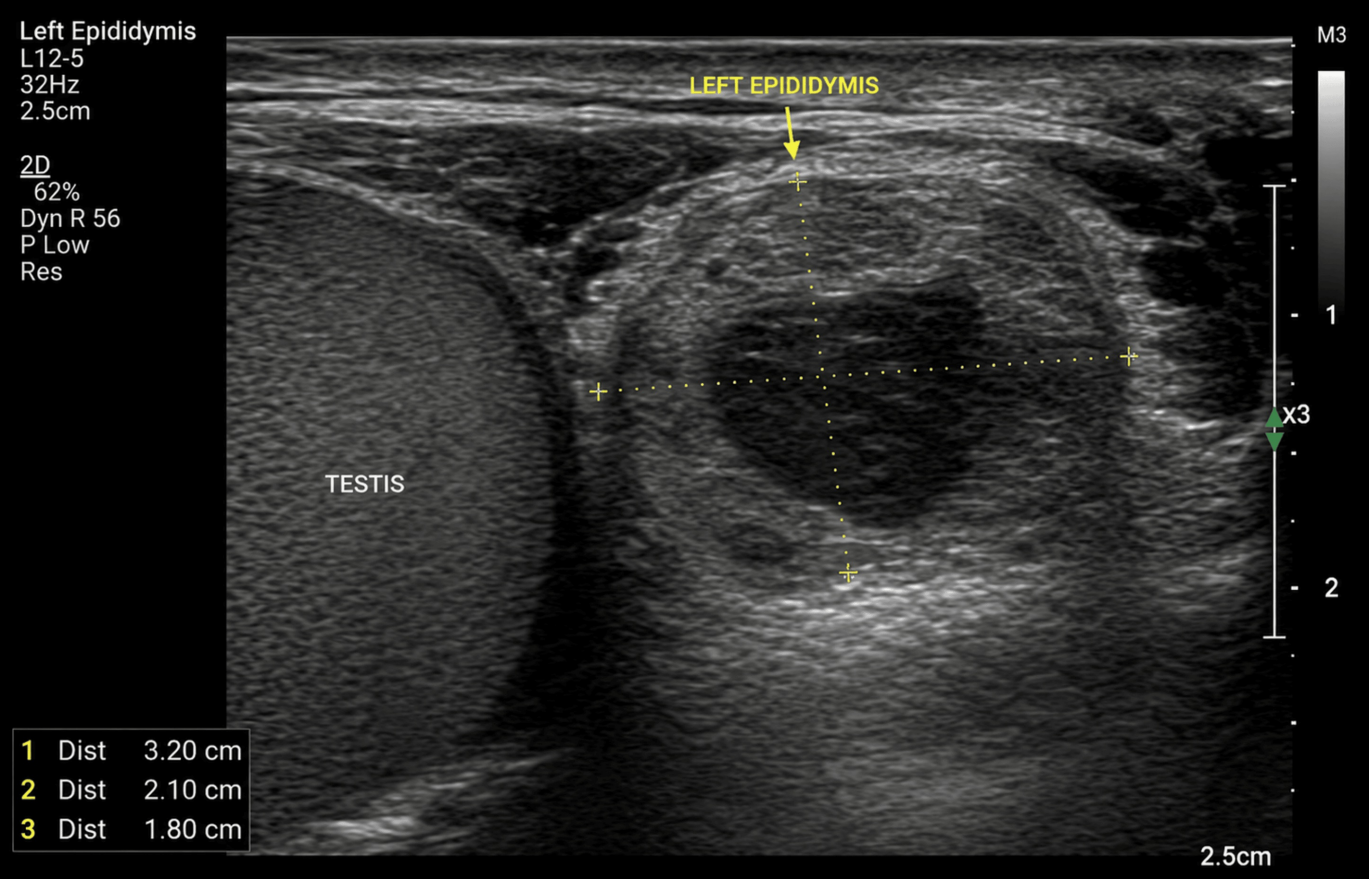
Thickening of the left epididymis measuring 3.2 × 2.1 × 1.8 cm, with a heterogeneous appearance and a hypoechoic center, consistent with an epididymal abscess

A 36-year-old man with no significant past medical or surgical history presented to the urology department of the Military Hospital Oued Eddahab in Agadir, Morocco, with a two‑month history of progressive left scrotal pain associated with intermittent fever. The patient did not report urinary symptoms, trauma, or prior tuberculosis. On examination, he was febrile (39°C). Local examination revealed a painful swelling of the left hemiscrotum with erythema and fluctuation suggestive of a scrotal abscess. The contralateral testis was normal, and no inguinal lymphadenopathy was detected. Digital rectal examination revealed a soft, non-tender prostate. A marked inflammatory syndrome was noted on laboratory testing, with leukocytosis (15,300/mm³) and elevated C-reactive protein (125 mg/L). Serological screening for human immunodeficiency virus, hepatitis B virus, and hepatitis C virus was negative. Scrotal ultrasound revealed a thickening of the left epididymis measuring 3.2 × 2.1 × 1.8 cm, with a heterogeneous appearance and a hypoechoic center, consistent with an epididymal abscess (Figure 1). No vascular compromise of the testis was observed, and the contralateral epididymis and testis were normal. Thoracic imaging showed no evidence of active pulmonary tuberculosis.

The patient initially underwent surgical drainage of a presumed scrotal abscess under general anesthesia. A scrotal incision was made, allowing evacuation of purulent fluid. The abscess cavity was carefully explored, and thorough irrigation with saline solution was performed. Limited debridement of necrotic and inflamed tissue was performed. A drain was placed, and the wound was managed according to standard principles. The patient was started on broad-spectrum intravenous antibiotics.

**Figure 2:**
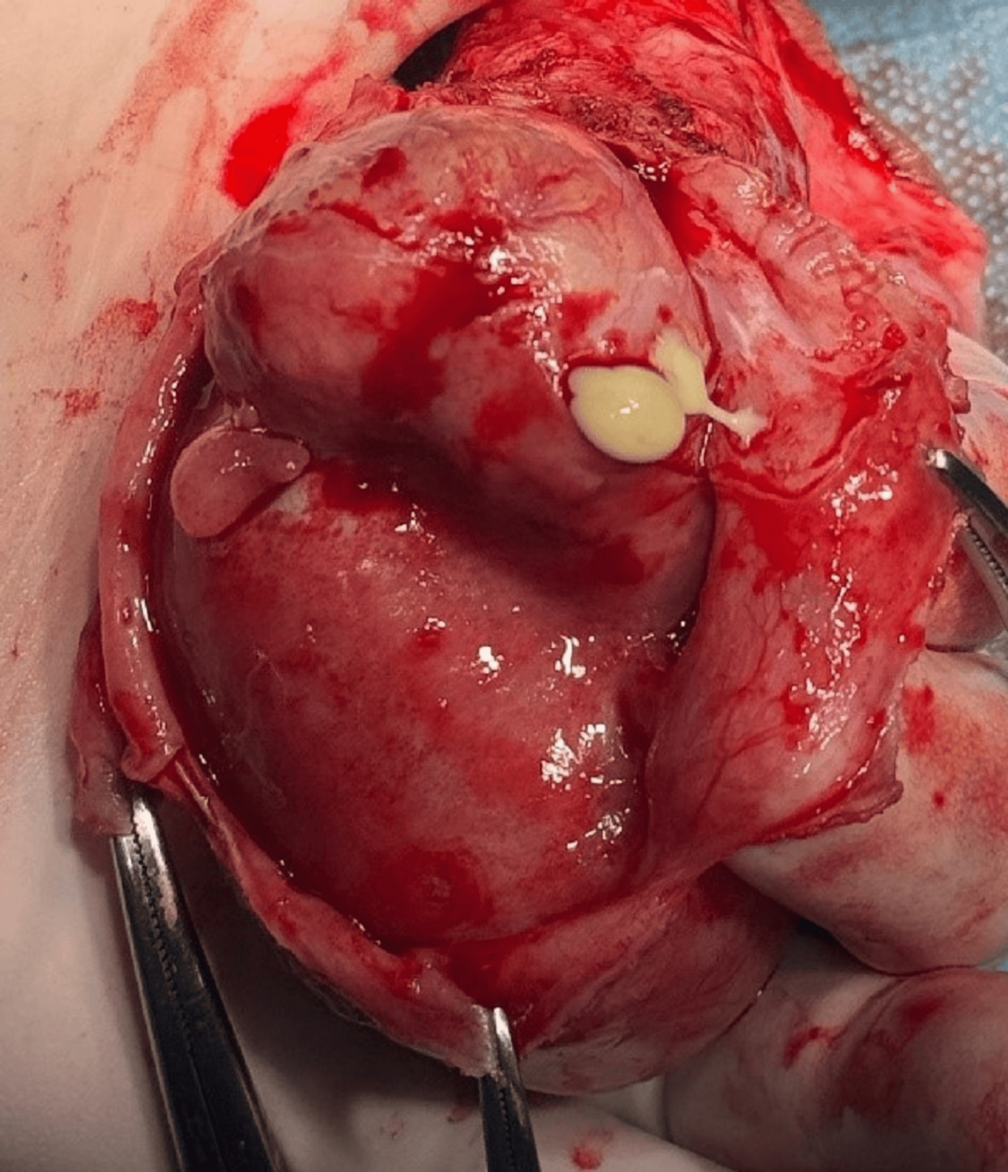
Intraoperative view showing scrotal abscess and necrotic tissue before surgical debridement

Ten days later, due to persistent symptoms, including pain, fever, and ongoing local inflammatory signs, a second surgical exploration was performed. Intraoperative findings revealed progressive necrotic changes of the scrotal tissues. Extensive debridement of all non-viable tissue was undertaken until healthy bleeding margins were obtained. Careful inspection of the scrotal contents confirmed that the epididymis was preserved, with no evidence of resection or injury. The testes were also viable. The surgical field was thoroughly irrigated, hemostasis was secured, and a drain was repositioned. The wound was left to heal according to the extent of local contamination (Figure 2).

**Figure 3:**
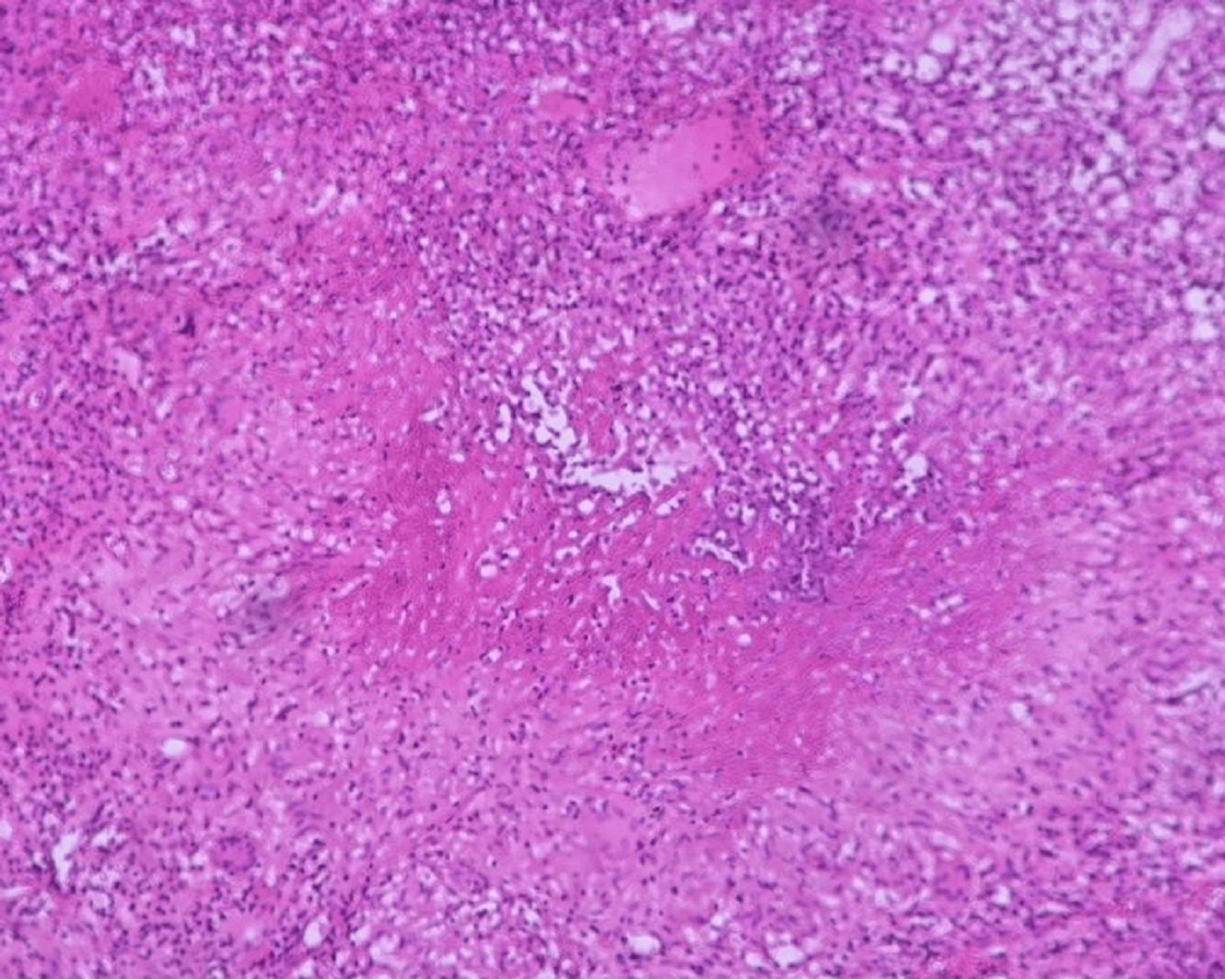
Histopathological image showing granulomatous inflammation with caseous necrosis consistent with tuberculous infection

Direct smear microscopy of the drained pus revealed acid‑fast bacilli. Culture subsequently confirmed *Mycobacterium tuberculosis*, and polymerase chain reaction testing for the *Mycobacterium tuberculosis* complex was positive. The anatomopathology of the excision was consistent with granulomatous inflammation with caseous necrosis, suggesting a tuberculous infection (Figure 3).

Once the diagnosis was confirmed, anti-tuberculosis therapy was initiated according to the standard six-month regimen, with weight-adjusted dosages: isoniazid 5 mg/kg/day (not exceeding a daily dose of 300 mg), rifampicin 10 mg/kg/day (not exceeding 600 mg/day), pyrazinamide 20-25 mg/kg/day (not exceeding 2 g/day), and ethambutol 15-20 mg/kg/day (not exceeding 1600 mg/day) for two months (induction phase), followed by isoniazid and rifampicin at the same weight-adjusted doses for four months (maintenance phase). Pyridoxine (25-50 mg/day) was also administered.

The patient underwent regular clinical and laboratory follow-up to detect potential adverse effects, including liver and ocular function assessments to screen for ethambutol-related optic neuropathy. No clinically significant adverse events necessitating treatment modification were observed. The patient had a favorable outcome with progressive resolution of scrotal inflammation and remained well without recurrence at 12‑month follow‑up.

## Discussion

GUTB remains an important cause of extrapulmonary tuberculosis, particularly in endemic settings, and continues to be associated with diagnostic delay because of its protean and frequently nonspecific presentation [1-4]. Within male genital tuberculosis, the epididymis is among the most commonly affected structures; infection is typically the result of hematogenous spread from a primary focus, although contiguous or retrograde spread from the urinary tract has also been described [2-4]. Clinical manifestations of epididymal tuberculosis are often nonspecific and can mimic common scrotal diseases. An initial diagnosis of epididymo-orchitis is most often made based on patient complaints such as a painful swollen scrotum, a palpable epididymal nodule, or even induration in advanced stages. Empirical antibiotic therapy is then usually initiated [2-4]. This atypical presentation often leads to a delayed diagnosis, thus exposing patients to unnecessary investigations. Abscess formation is an uncommon but recognized complication of epididymal tuberculosis. Chronic granulomatous inflammation may lead to caseous necrosis and liquefaction, producing a collection that can resemble a pyogenic abscess or a tumor-related process [5]. Recent case reports continue to highlight that epididymal or scrotal abscesses may be the first manifestation of genital tuberculosis and that low clinical suspicion can result in repeated drainage and orchidectomy before tuberculosis is considered [5].

Routine laboratory findings (leukocytosis, elevated C-reactive protein) are nonspecific. The diagnosis is based on a combination of bacteriological, molecular, and histopathological findings. Direct examination of a smear for acid-fast bacilli can quickly provide presumptive evidence. However, its sensitivity remains variable in extrapulmonary locations. Culture remains the reference standard, but time to results can be prolonged. WHO-endorsed rapid molecular tests (nucleic acid amplification tests) have improved early detection in extrapulmonary tuberculosis and can support timely initiation of therapy when clinical suspicion is high [6]. Histopathology remains highly valuable: granulomatous inflammation with caseous necrosis strongly supports a diagnosis of tuberculosis, especially in endemic regions, and in our patient it was consistent with the microbiological findings. Ultrasonography is the first-line imaging modality for scrotal pathology and can demonstrate epididymal enlargement, heterogeneous echotexture, hydrocele, calcifications, or abscesses, but these findings are not pathognomonic [2-4]. When available, MRI may provide superior soft-tissue characterization and may help radiologists and urologists recognize patterns suggestive of epididymal and testicular tuberculosis [7]. The cornerstone of treatment is anti-tuberculosis therapy. The WHO currently recommends a standard treatment regimen for urogenital tuberculosis. This regimen consists of an intensive phase with isoniazid, rifampicin, pyrazinamide, and ethambutol, followed by a maintenance phase with isoniazid and rifampicin. Dose adjustments are possible depending on the clinical context [8]. Surgery should be reserved for cases of medical treatment failure of medical treatment, complications (such as abscess, fistula, or extensive necrosis), or diagnostic uncertainty [2-4]. In our case, the suspicion of a pyogenic abscess necessitated surgical drainage with wide debridement. However, definitive management required bacteriological confirmation and the rapid initiation of anti-tuberculosis therapy, resulting in a favorable clinical outcome.

This case underlines the importance of considering epididymal tuberculosis in patients with chronic or recurrent scrotal abscesses, particularly in tuberculosis-endemic areas. Early sampling for mycobacterial microscopy, culture, and rapid molecular testing, together with histopathological assessment when tissue is available, can shorten the diagnostic pathway, reduce unnecessary antibiotic exposure and repeat procedures, and prevent complications.

Differential diagnosis includes pyogenic epididymo-orchitis with scrotal abscess, testicular or paratesticular malignancy, brucellosis, fungal infections, and other granulomatous diseases (e.g., sarcoidosis). In endemic settings, tuberculosis should be considered early in chronic or recurrent scrotal suppuration, particularly when response to standard antibiotics is suboptimal.

## Conclusions

Epididymal tuberculosis is an uncommon but important cause of chronic scrotal infection. Its presentation as a scrotal abscess may lead to diagnostic delay and repeated surgical procedures. Urogenital tuberculosis should be suspected in patients with a scrotal abscess that does not respond to standard antibiotic therapy, particularly in endemic areas. A favorable prognosis is closely linked to early diagnosis and appropriate anti-tuberculosis therapy.
